# Natural history, response to systemic therapy, and genomic landscape of plasmacytoid urothelial carcinoma

**DOI:** 10.1038/s41416-020-01244-2

**Published:** 2021-01-21

**Authors:** Min Yuen Teo, Hikmat Al-Ahmadie, Kenneth Seier, Christopher Tully, Ashley M. Regazzi, Eugene Pietzak, David B. Solit, Satish Tickoo, Victor Reuter, Eugene K. Cha, Harry Herr, Timothy Donahue, Sherri M. Donat, Guido Dalbagni, Bernard H. Bochner, Samuel Funt, Gopakumar V. Iyer, Dean F. Bajorin, Irina Ostrovnaya, Jonathan E. Rosenberg

**Affiliations:** 1 Department of Medicine, Memorial Sloan Kettering Cancer Center, New York, NY USA; 2 Department of Pathology, Memorial Sloan Kettering Cancer Center, New York, NY USA; 3 Department of Epidemiology & Biostatistics, Memorial Sloan Kettering Cancer Center, New York, NY USA; 4 Morristown Medical Center, Morristown, NJ USA; 5 Department of Surgery, Urology Service, Memorial Sloan Kettering Cancer Center, New York, NY USA; 6 Marie-Josée and Henry R. Kravis Center for Molecular Oncology, Memorial Sloan Kettering Cancer Center, New York, NY USA; 7 Weill Cornell Medical College, New York, NY USA

**Keywords:** Outcomes research, Bladder cancer

## Abstract

**Background:**

Plasmacytoid urothelial carcinoma (PUC) is a rare, aggressive histologic variant of urothelial cancer characterised by a diffuse growth pattern and *CDH1* mutation. We studied the efficacy of preoperative platinum-based chemotherapy in nonmetastatic PUC and immune checkpoint inhibitors (ICIs) in advanced PUC.

**Methods:**

Cases of nonmetastatic PUC and advanced PUC treated with ICIs at our institution were identified. Outcomes were compared to those of a published cohort of patients with urothelial carcinoma not otherwise specified.

**Results:**

We identified 81 patients with nonmetastatic PUC. Of the patients with localised disease who underwent neoadjuvant chemotherapy, pathologic complete response and downstaging rates were 12 and 21%, respectively. Pathologic downstaging was not associated with significant improvement in clinical outcomes. Up to 18% of localised disease and 28% of locally advanced cases had unresectable disease at the time of surgery. ICI-treated advanced PUC (*N* = 21) had progression-free and overall survival of 4.5 and 10.5 months, respectively, and a 38% response rate. *FGFR3* and DNA damage response gene alterations were observed in 3 and 15% of cases, respectively.

**Conclusions:**

PUC is associated with high disease burden and poor chemosensitivity. Increased awareness and recognition of this disease variant will allow for new treatment strategies.

## Background

Plasmacytoid urothelial carcinoma (PUC) is a rare histologic variant of urothelial carcinoma (UC) characterised by an aggressive natural history and clinical course.^1^ It has a diffuse growth pattern^2^ and is more likely to be locally advanced at diagnosis than conventional UC (not otherwise specified [NOS]).^3^ Somatic truncating alterations in *CDH1*, which encodes the protein E-cadherin, have been reported in 80% of sequenced PUC cases; hypermethylation of the *CDH1* promoter is found in other PUC cases.^4^ Although some data have suggested that PUC survival outcomes are not inferior to UC NOS when matched for disease stage at diagnosis, the rate of perioperative chemotherapy use is low in these analyses.^5,6^

Preoperative cisplatin-based chemotherapy increases pathologic responses and cancer-specific survival^7^ and is an integral component of modern management of UC. The presence of variant histology does not appear to compromise the efficacy of preoperative chemotherapy in patients with UC of squamous or glandular differentiation.^8,9^ However, the utility of preoperative chemotherapy for patients with PUC remains poorly defined. Anti-programmed cell death 1 (anti-PD1)/programmed cell death ligand 1 (PDL1) immune checkpoint inhibitors (ICIs) are also used to treat patients with UC^10–13^ and have an emerging role in preoperative therapy.^14,15^ However, the efficacy of these agents in PUC has not been studied.

In describing our institutional experience in the management of PUC, the primary objectives of this study are to (1) define the clinicopathologic features of PUC at diagnosis, (2) determine the response of PUC to preoperative platinum-based chemotherapy, and (3) further describe the genomic landscape of this under-studied disease entity. We also described the clinical activity of ICIs in a cohort of patients with advanced PUC.

## Methods

**Fig. 1:**
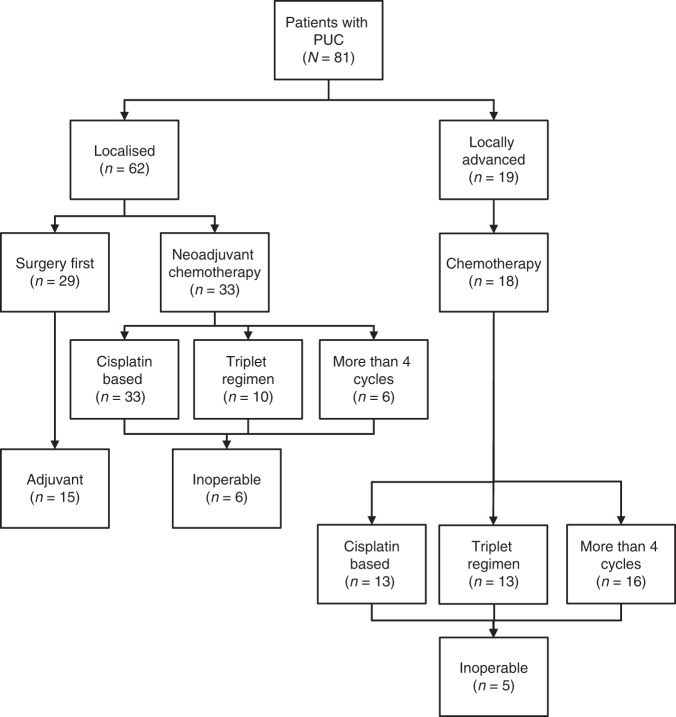
Consort diagram depicting the study population and subgroups for the primary clinical analysis. PUC plasmacytoid urothelial carcinoma.

This work was performed under an institutional review board–approved protocol. Cases of PUC of the bladder diagnosed at our institution were identified, and all cases were reviewed by a genitourinary pathologist (H.A.-A.) to confirm the diagnosis. Cases diagnosed between 2000 and 2017 with clinical follow-up were included for primary clinical analysis (Fig. 1). Only cases that were nonmetastatic at presentation were included in the study. All cases were classified as either localised PUC (cT2–T4a N0 disease) or locally advanced PUC (radiographic evidence of regional lymphadenopathy and/or fixed bladder at examination under anaesthesia). Tumours were classified according to the 2016 World Health Organisation Classification of Tumours of the Urinary System.^1^ Tumours were characterised by a diffuse and discohesive growth pattern with minimal stroma reaction. Tumour cells had eccentric or centrally located nuclei. Cytoplasmic vacuoles and signet ring cells were identified in all cases at variable proportions.

Pathologic stages were compared between patients with clinically localised PUC who underwent either neoadjuvant chemotherapy before cystectomy or cystectomy first. Outcomes were also compared between patients with PUC and patients with UC NOS. A cohort of patients with UC NOS (*N* = 146) treated with neoadjuvant cisplatin and gemcitabine for whom clinical outcome data have been reported was used as a comparator for this analysis.^16^

An expanded pathology database of cases of PUC diagnosed between 2000 and 2020 was used to identify cases that were genomically profiled (Supplementary Fig. S1). Tumours were sequenced using Memorial Sloan Kettering Integrated Mutation Profiling of Actionable Cancer Targets (MSK-IMPACT), a Food and Drug Administration-authorised, hybridisation capture–based, next-generation sequencing platform.^17,18^ Genes that are sequenced in MSK-IMPACT were chosen based on documentation in the Catalogue of Somatic Mutations in Cancer^19,20^ and annotation of oncogenicity in the OncoKB Precision Oncology Knowledge Base (https://oncokb.org).^21^ Alterations in DNA damage response (DDR) genes were also evaluated. DDR status was classified based on the presence of mutations in 34 genes that are involved in different DDR pathways and are in the MSK-IMPACT sequencing panel. The expanded database was also used to identify patients with advanced PUC who received anti-PD1/PDL1 ICIs.

Fisher’s exact and Wilcoxon rank-sum tests were used to test the significance of differences of categorical and continuous data, respectively. Recurrence-free survival (RFS) was calculated from the date of radical cystectomy (or attempted surgery) to the date of radiographic evidence of recurrence or death. Patients whose cystectomies were aborted were excluded from RFS analysis. Overall survival (OS) was calculated from the date of radical cystectomy (or attempted surgery) to the date of death. Survival was estimated by Kaplan–Meier and compared by log-rank test. Cox proportional hazard models were used for univariate and multivariate analyses, and interactions were tested between histology (PUC vs UC NOS) and response (responders vs non-responders). Analyses were conducted with SAS version 9.4 (SAS institute Inc., Cary, NC). All tests were two sided, and *p* < 0.05 was considered significant.

## Results

We identified 81 patients with nonmetastatic PUC (Fig. 1) who received treatment at our institution. The majority of patients (*n* = 62, 77%) had clinically localised disease; 19 (23%) patients had locally advanced disease at diagnosis, defined as the presence of fixed tumour at examination under anaesthesia or unequivocal radiographic evidence of locoregional lymphadenopathy. Baseline characteristics of the study population are listed in Table 1.

**Table 1:** Patient and clinical characteristics for plasmacytoid urothelial carcinoma and urothelial carcinoma not otherwise specified cohorts.

	All PUC	Up-front surgery PUC	NAC PUC	LA PUC	UC NOS
	(*N* = 81)	(*N* = 29)	(*N* = 33)	(*N* = 18)	(*N* = 146)
Age					
Median (range)	65 (22–84)	50 (43–84)	59 (43–76)	64 (22–84)	65 (39–82)
Sex					
Male	53 (65)	19 (66)	25 (76)	8 (44)	101 (46)
Female	28 (35)	10 (35)	8 (24)	10 (56)	45 (31)
Clinical T stage					
T2	57 (70)	28 (97)	23 (70)	5 (27.8)	67 (45.9)
T3–4	24 (30)	1 (3)	10 (30)	13 (72.2)	79 (54.1)
Clinical N stage					
Negative	73 (90)	29 (100)	33 (100)	8 (44.4)	146 (100)
Positive	8 (10)	0 (0)	0 (0)	10 (55.6)	0 (0)
EUA findings					
Normal	50 (62)	14 (48)	30 (91)	6 (33)	N/A
LA/invasive	12 (15)	0 (0)	0 (0)	11 (61)	N/A
ND/NP	19 (24)	15 (52)	3 (9)	1 (6)	N/A
Preoperative chemotherapy					
Yes	51(63)	0 (0)	33 (100)	18 (100)	146 (100)
No	30 (37)	29 (100)	0 (0)	0 (0)	0 (0)
Platinum type					
Cisplatin based	46 (90)	N/A	33 (100)	13 (72)	146 (100)
Carboplatin based	5 (10)	N/A	0 (0)	5 (28)	0 (0)
Duration of systemic therapy					
4 cycles	29 (57)	N/A	27 (82)	2 (11)	146 (100)
>4 cycles	22 (43)	N/A	6 (18)	16 (89)	0 (0)

The patient and clinical characteristics of the patients in the study are shown. Values represent frequency (percentage) unless specified otherwise.

*PUC* plasmacytoid urothelial carcinoma, *NAC* neoadjuvant chemotherapy, *LA* locally advanced, *UC NOS* urothelial carcinoma not otherwise specified, *EUA* examination under anaesthesia, *N/A* not applicable, *ND/NP* not documented/not performed.

### Clinically resectable, localised PUC

Of the 62 patients with localised muscle-invasive PUC, 29 (47%) underwent surgery first. Thirty-three (53%) patients received neoadjuvant chemotherapy, all of whom received cisplatin-based chemotherapy. Pathologic staging of these patients is shown in Table 2 and Supplementary Table S1. Although 97% of those who underwent up-front surgery had radiographic cT2 disease, 79% had pT3–4 disease on pathologic evaluation, and 38% had positive nodes. Twelve (41%) of the 29 patients in the surgery only group received adjuvant chemotherapy, of which 11 (92%) had pT3/4 disease and 6 (50%) had nodal involvement. In comparison, 12 (71%) of the 17 patients who did not receive adjuvant chemotherapy had pT3/4 disease and 5 (29%) had nodal involvement. (*p* = 0.06 for pT3–4 disease; *p* = 0.44 for nodal involvement).

**Table 2:** Pathologic staging of patients with clinically localised plasmacytoid carcinoma treated with neoadjuvant chemotherapy or surgery first.

	Neoadjuvant	Surgery first	*p* value
	(*N* = 33)	(*N* = 29)	
Pathologic T stage			
<pT2	7 (21)	2 (7)	0.048
pT2–4	26 (79)	27 (93)	
Pathologic N stage			
Node negative	19 (58)	18 (62)	0.018
Node positive	7 (21)	11 (38)	
Not available	7 (21)	0 (0)	
AJCC staging			
0–I	7 (21)	2 (7)	0.155
II–IV	26 (79)	27 (93)	

Values represent frequency (percentage).

*AJCC* American Joint Committee on Cancer.

Of the patients who received neoadjuvant chemotherapy, 10 (30%) patients received triplet chemotherapy regimen (cisplatin, gemcitabine, and paclitaxel), and 6 (18%) patients received more than four cycles of chemotherapy (up to six planned cycles). Cystectomy was aborted in 6 (18%) cases at the time of surgery due to locally advanced, unresectable disease following chemotherapy. All six patients had fixed bladders—three to the pelvic side wall, two to the intestines and/or rectum, and one to both. Three of these patients had positive biopsies that confirmed disseminated disease.

Following neoadjuvant chemotherapy, pathologic downstaging to non-muscle-invasive disease (<ypT2 ypN0) was observed in 7 (21%) patients, 4 (12%) of which had pathologic complete response. Of the remaining three patients, two had carcinoma in situ and one had pT1 disease. Triplet chemotherapy, extended number of chemotherapy cycles, or other clinical variables were not associated with response to neoadjuvant chemotherapy (Supplementary Table S2). Of the 23 patients with cT2 disease at diagnosis, 10 (43%) had ypT3–4 disease and 6 (26%) had nodal involvement at pathologic evaluation despite neoadjuvant chemotherapy. Three surgeries were aborted due to fixed bladder at attempted cystectomy.

### Locally advanced PUC

Locally advanced PUC was defined as disease with regional lymphadenopathy and/or fixed tumour on examination under anaesthesia. Nineteen patients with locally advanced disease were identified, 18 of whom received systemic chemotherapy. The patient who did not receive systemic therapy underwent up-front surgery. Post-surgical pathology showed extensive bladder wall involvement along the entire circumference, with extensive invasion into the perivesical soft tissue, prostate, and seminal vesicles and positive surgical margins. Local recurrence was noted 4.6 months later.

Among those who received systemic therapy, 10 (56%) had locally advanced disease per examination under anaesthesia, 7 (39%) had radiographic evidence of regional lymphadenopathy, and 1 had both features. Most patients received cisplatin (*n* = 13, 72%), triplet chemotherapy regimen (*n* = 13, 72%), and >4 cycles of chemotherapy (*n* = 16, 89%). Although resection was attempted for every patient with locally advanced PUC after chemotherapy, the surgeries of 5 (28%) patients were aborted due to inoperable disease at intraoperative assessment.

Four (22%) patients had pathologic responses, with 2 (11%) pathologic complete responses. Median RFS and OS for patients with locally advanced PUC were 5.7 and 10.6 months, respectively, with 19% of patients alive at 2 years. The OS of patients whose tumours were found unresectable during surgery was significantly shorter than the OS of those who underwent resection (6.0 and 19.6 months, respectively; hazard ratio [HR] 3.57; 95% confidence interval [CI] 0.76–16.65, *p* = 0.009).

### Comparison of PUC and UC NOS clinical outcomes

A previously published cohort of patients with muscle-invasive UC NOS of the bladder treated with neoadjuvant cisplatin and gemcitabine (*n* = 146) was used as comparator for RFS and OS.^16^ The percentage of patients with pathologic complete response was lower in patients with PUC as compared to patients with UC NOS, but this difference was not statistically significant (12 vs 21%, *p* = 0.456). The percentage of patients with pathologic downstaging (<ypT2 pN0) was significantly lower in patients with PUC than in patients with UC NOS (21 vs 45%, *p* = 0.018).

Patients with PUC treated with neoadjuvant cisplatin-based chemotherapy had significantly inferior RFS and OS than patients with UC NOS who received neoadjuvant chemotherapy (median RFS 16.7 months vs not reached [NR], log-rank *p* < 0.001; median OS 30.2 months vs NR, log-rank *p* < 0.004).

**Fig. 2:**
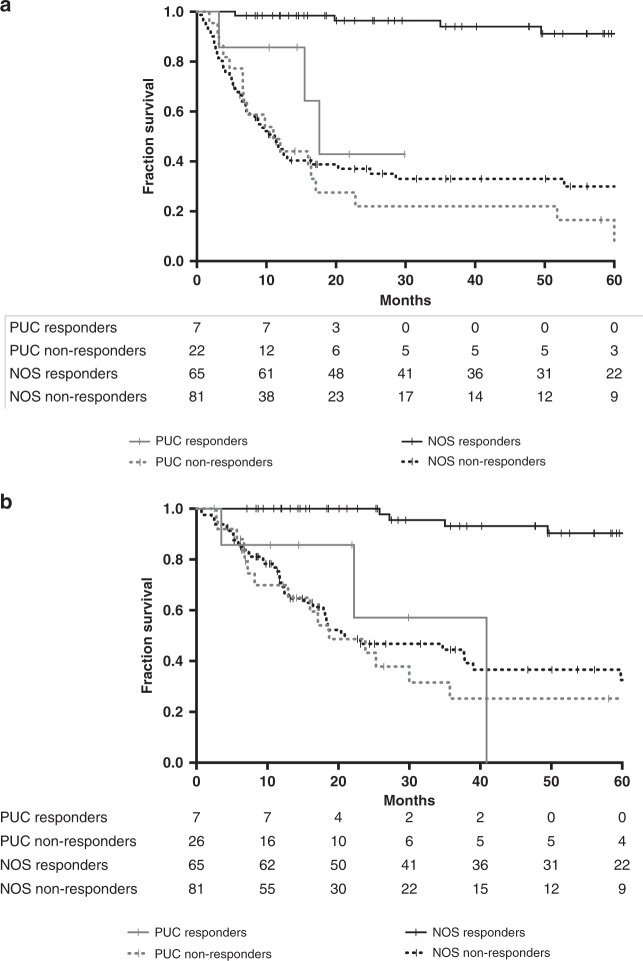
Recurrence-free and overall survival is reduced in plasmacytoid urothelial carcinoma. Recurrence-free survival (**a**) and overall survival (**b**) of patients with plasmacytoid urothelial carcinoma (PUC) and urothelial carcinoma, not otherwise specified (UC NOS) treated with neoadjuvant chemotherapy. Survival is compared between responders (those with pathologic complete response, defined as <pT2 pN0 disease on pathologic evaluation) and nonresponders.

RFS and OS were also compared between PUC and UC NOS based on pathologic responses (Fig. 2a, b). For responders, PUC patients had worse RFS (HR 10.17, 95% CI 2.26–45.72) and OS (HR = 11.47, 95% CI 2.55–51.57). For non-responders, RFS (HR = 1.16, 95% CI 0.66–2.03) and OS (HR = 1.20, 95% CI 0.66–2.16) were comparable.

One of the 4 patients with pathologic complete response experienced disease recurrence after 15.5 months, with an overall survival of 22.2 months. The other three remained recurrence-free after 10.4, 14.4, and 29.9 months of follow-up. Of the 3 patients with residual non-muscle invasive disease, 2 (67%) had relapses: one at 3.2 months, who died after 3.5 months, and another at 17.6 months, who died after 40.9 months. The third patient remained recurrence-free at 21.9 months follow-up.

### Patterns of failure

Of the 29 patients with clinically localised PUC treated with cystectomy first, 11 (38%) patients had documented recurrences—5 (45%) osseous, 4 (36%) nodal, 2 (18%) peritoneal, and 1 (9%) pelvic. One patient had two sites of disease at first recurrence. Of the 4 nodal recurrences, 3 (75%) were retroperitoneal lymphadenopathy.

Of the patients with clinically localised PUC who received neoadjuvant chemotherapy, 17 patients (52%) had documented recurrences—sites of recurrence included: 4 (24%) nodal (all retroperitoneal), 4 (24%) peritoneal, 4 (24%) local, 3 (18%) osseous, 2 (12%) hepatic, 2 (12%) pulmonary, and 1 (6%) brain.

Of those with locally advanced disease treated with chemotherapy, cystectomy was aborted in 5 (28%) cases. The most common patterns of failure were peritoneal (7 patients, 39%), osseous (2 patients, 11%), nodal (1 patient, 6%), hepatic (1 patient, 6%), and pulmonary (1 patient, 6%). Three cases of peritoneal recurrence presented as bowel obstruction.

### Anti-PD1/PDL1 immune checkpoint blockade in advanced PUC

Between September 2014 and May 2020, 21 patients with advanced PUC were treated with ICIs (19 with anti-PD1/PDL1 monotherapy and 2 with anti-PD1 and anti-CTLA4 combination). Baseline characteristics are tabulated in Supplementary Table S3.

Among the 19 patients with advanced PUC, 6 (32%) experienced radiographic response. Median progression-free survival and OS were 4.5 and 10.5 months, respectively (Supplementary Fig. S1). Median duration of response was 17.0 months, with 2 on-going responses at 26.0 and 66.5 months at the time of analysis.

The two patients who received combination therapy had on-going responding disease for 4.5 and 7.2 months at the time of data analysis. Responses were observed in 8/21 patients (38%, including both patients treated with combination therapy).

### Genomic characterisation of PUC

Thirty-three tumours in our database of 122 pathologically reviewed and confirmed cases of PUC were sequenced using MSK-IMPACT (Supplementary Fig. S2). None of these tumours had been sequenced in our previous analysis.^4^ Deleterious *CDH1* alterations were noted in 20/33 (61%) cases.

Median tumour mutation burden (TMB) for the cohort was 14.9 mutations/Mb (range 1.8–45.6; interquartile range 8.9–21.6); this represents the 75th percentile among all UC (UC NOS and variant histologies) in our MSK-IMPACT database (*N* = 1961 samples as of June 2020). For the ICI-treated subset, 16/21 (76%) had MSK-IMPACT performed, with median TMB of 18.4 mutations/Mb (range 3.3–45.6; interquartile range 8.9–26.6). The median TMB for ICI monotherapy responders and non-responders was 15.3 and 14.3 mutations/Mb, respectively (*p* = 0.8).

Alterations in DDR genes have been shown to be associated with platinum responsiveness.^22,23^ Five (15%) tumours harboured six alterations in DDR genes: *ERCC2* (I174M and I444M), *ATM* (E38*, K196*, and E2904Dfs*29), *BRCA2* (E23Vfs*17), and *RECQL4* (X822_splice). Neither *ERCC2* alteration has been functionally validated, but the mutated residues are at or near the helicase domains.

**Fig. 3:**
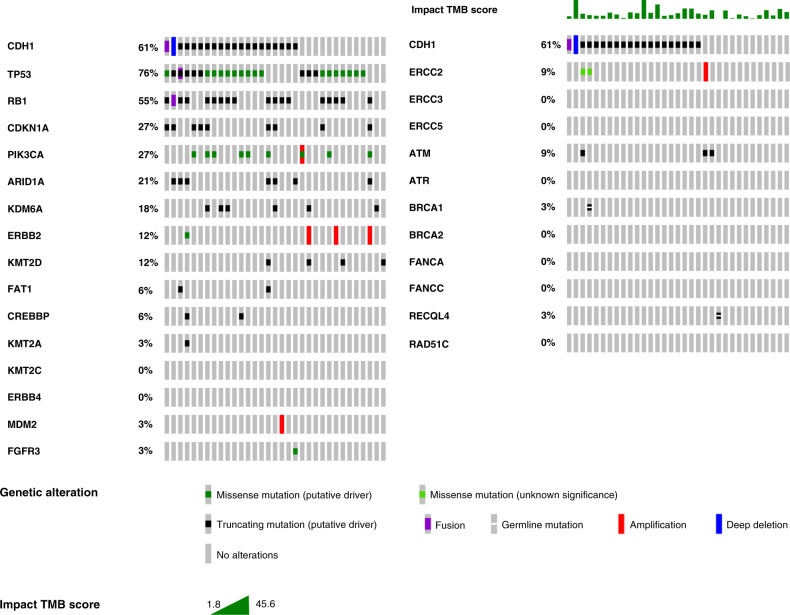
Genomic characterisation of plasmacytoid urothelial carcinoma. Oncoprint of 33 plasmacytoid urothelial carcinoma cases. Left, the prevalence of alterations in genes commonly altered in urothelial carcinoma, not otherwise specified, per The Cancer Genome Atlas analysis. Right, the prevalence of alterations in selected DNA damage response genes.

Although the genes mutated in PUC were not dissimilar to those mutated in UC NOS,^24^ we observed higher rates of alterations in *TP53* (25 cases, 76 vs 48%, *p* = 0.006) and *RB1* (18 cases, 55 vs 17%, *p* < 0.001) compared to UC NOS from The Cancer Genome Atlas data set.^25^ Only 1 (3%) case in the PUC cohort harboured an alteration in *FGFR3* (Fig. 3).

## Discussion

PUC is an aggressive variant of UC that is characterised by poor clinical outcomes and is frequently locally advanced at diagnosis. Our series represents one of the largest efforts in evaluating the natural history and clinicopathologic characteristics of nonmetastatic PUC. This is also the largest series examining the response of PUC to preoperative platinum-based chemotherapy and the first assessment of response of advanced PUC to ICIs. For clinically resectable PUC, administration of cisplatin-based chemotherapy was associated with lower pathologic compete response and pathologic downstaging rates than UC NOS. Although many patients receive neoadjuvant chemotherapy, survival among patients with PUC was significantly lower than for patients with UC NOS, even among pathologic responders. Even after preoperative chemotherapy, a large proportion of PUCs were found to be unresectable at the time of surgery. Conversely, response rates and clinical outcomes with ICIs in advanced disease were not dissimilar to reported studies in urothelial cancers in general.^10–13^

The unique pattern of PUC dissemination contributes to the under-appreciation of disease extent. In our series, extravesical disease was prevalent among patients who underwent cystectomy first, and cystectomy was aborted due to extensive and fixed disease in 18–28% of patients who underwent preoperative chemotherapy, despite the absence of radiographic progression after systemic therapy. This is consistent with prior reports indicating that PUC is characterised by sheet-like peritoneal dissemination^26^ and is associated with a higher probability of margin involvement.^3,5^ In accordance with other published series,^27^ we reported a high number of recurrences, which were frequently in the peritoneum.

PUC might also be associated with reduced chemosensitivity, as we observed decreased pathologic response and survival than in UC NOS. A post hoc analysis from the AUO-AB05/95 adjuvant trial^28^ and other retrospective series^27,29^ did not show a survival advantage for preoperative platinum-based chemotherapy over cystectomy alone. In a recently reported series of 26 PUC cases, 2 pathologic complete responses (10%) and 1 residual non-muscle invasive disease (5%) were noted, an observation consistent with our findings.^30^ We also observed a low rate of genomic alterations in DDR genes, including *ERCC2* and *ATM*, which have been reported to be associated with response to platinum-based chemotherapy.^22,23,29,31,32^

Treatment with anti-PD1/PDL1 ICI was associated with durable benefit in a third of patients in our series. Our observed response rate was comparable to ICI data from trials of patients with metastatic UC,^10,33^ but contrasts with a series of 13 patients with advanced PUC treated with ICI who had poor survival.^34^ More recently, an interim analysis of a Phase 2 trial of rare genitourinary cancers reported promising activity with ipilimumab and nivolumab, although only one PUC case was enrolled and responded to treatment at the time of reporting.^35^

Our genomic analysis confirmed the high rate of truncating *CDH1* alterations in PUC and further characterised other genes that are frequently altered in PUC. The current series represents the largest cohort of genomically characterised PUC and is independent from our previous analysis, which reported truncating *CDH1* mutations in 26/31 (84%) cases.^4^ Compared with published UC NOS sequencing data, we observed higher rates of alterations in *TP53* and *RB1* and lower rates of alterations in *FGFR3* and DDR genes, which is consistent with other PUC series.^4,36^

Our study was limited by its size, single institutional, and retrospective nature, with patients treated over a 20-year period. This is unfortunately inevitable due to the rarity of PUC. During this 17-year period, different histomorphological definitions existed; these were updated and consolidated recently.^1^ To ensure that all cases that were included in our study were PUC, a detailed histopathologic review was performed on each case to confirm the diagnosis.

In summary, in our series of pathologically verified PUC, preoperative chemotherapy response and clinical outcomes were poor relative to UC NOS, indicating that therapeutic strategies other than platinum-based chemotherapy are urgently needed. Genomic analyses have not identified potential therapeutic targets. We found a higher TMB in our cohort; this observation, in addition to PDL1 being expressed in 21–36% of cases, suggests a potential susceptibility to anti-PD1/PDL1 checkpoint blockade.^37^ In fact, in our small cohort of patients with advanced PUC, durable responses were observed in one-third of patients, supporting further investigations in the treatment of PUC with ICI. Indeed, the aforementioned Phase 2 trial of ipilimumab and nivolumab is still actively enrolling (NCT03333616). In fact, the Alliance A031702 Phase 2 trial of nivolumab, ipilimumab plus cabozantinib is a basket trial for rare genitourinary cancers, and it includes an arm for PUC (NCT03866382). Improved awareness of PUC as an aggressive disease entity is needed to ensure appropriate case selection in order to identify and refine therapeutic strategies for patients with PUC.

## Supplementary information

Supplemental Legends (for figures and tables) and Supplemental Tables

Supplemental Figures 1 and 2

## Data Availability

Anonymised data will be provided upon request.
