# Supplementary material for: Natural history, response to systemic therapy, and genomic landscape of plasmacytoid urothelial carcinoma
Source: Br J Cancer. 2021 Jan 21;124(7):1214–21. doi: 10.1038/s41416-020-01244-2 (PMC8007750; doi:10.1038/s41416-020-01244-2)
Supplement: Supplementary file 1 — Supplemental Legends (for figures and tables) and Supplemental Tables [file 41416_2020_1244_MOESM1_ESM.docx]

**SUPPLEMENTARY FIGURE AND TABLE LEGENDS**

**Supplementary Figure S1**: Consort diagram summarizing the Memorial Sloan Kettering plasmacytoid urothelial carcinoma database as of 12/2019. The distribution of tumors that were sequenced on the MSK-IMPACT tumor sequencing platform are shown. PUC, plasmacytoid urothelial carcinoma

**Supplementary Figure S2**: Progression-free and overall survival of PUC patients treated on anti-PD1/PDL1 immune checkpoint inhibitors. PUC, plasmacytoid urothelial carcinoma

**Supplementary Table S1**: Detailed staging of patients with clinically localized plasmacytoid carcinoma treated with neoadjuvant chemotherapy or surgery first. Numbers represent frequency (percentage).

**Supplementary Table S2:** Factors potentially affecting response to neoadjuvant chemotherapy. Values represent frequency (percentage) except when specified.

**Supplementary Table S3:** Characteristics of patients with advanced PUC treated with immune checkpoint inhibitors. Values represent frequency (percentage) unless otherwise indicated

| **Supplementary Table S1** Detailed staging of patients with clinically localized plasmacytoid carcinoma treated with neoadjuvant chemotherapy or surgery first. Numbers represent frequency (percentage). | | |
| --- | --- | --- |
|  |  |  |
|  | **Neoadjuvant** | **Surgery First** |
|  | **(*N* = 33)** | **(*N* = 29)** |
| **Pathologic T Stage** |  |  |
| pT0/pTis | 8 (24) | 1 (3) |
| pT1 | 1 (3) | 1 (3) |
| pT2 | 4 (12) | 4 (14) |
| pT3 | 11 (33) | 18 (62) |
| pT4 | 9 (27) | 5 (17) |
| **Pathologic N Stage** |  |  |
| Node negative | 19 (58) | 18 (62) |
| Node positive | 7 (21) | 11 (38) |
| Not available | 7 (21) | 0 (0) |
| **AJCC Staging** |  |  |
| 0/0s | 6 (18) | 1 (3) |
| I | 1 (3) | 1 (3) |
| II | 3 (9) | 2 (7) |
| III | 10 (30) | 14 (48) |
| IV | 13 (39) | 11 (38) |

| **Supplementary Table S2** Factors potentially affecting response to neoadjuvant chemotherapy. Values represent frequency (percentage) except when specified. | | | |
| --- | --- | --- | --- |
|  |  |  |  |
|  | **Responder (*N* = 7)** | **Nonresponder (*N* = 26)** | ***P* value** |
| **Age** |  |  |  |
| Median (range) | 60 (49–72) | 60 (41–76) | 0.809 |
| **Sex** |  |  |  |
| Male | 5 (71) | 20 (77) | >0.95 |
| Female | 2 (29) | 6 (23) |  |
| **Chemotherapy regimen** |  |  |  |
| Doublet chemotherapy | 4 (57) | 19 (73) | 0.646 |
| Triplet chemotherapy | 3 (43) | 7 (27) |  |
| **Duration of chemotherapy** |  |  |  |
| Up to four cycles | 5 (71) | 22 (85) | 0.584 |
| Extended, up to six cycles | 2 (29) | 4 (15) |  |

|  |  |
| --- | --- |
|  | **Advanced PUC treated with ICI (N = 21)** |
| **Age** |  |
| Median (range) | 66 (55–76) |
| **Sex** |  |
| Male | 13 (62) |
| Female | 8 (38) |
| **Neoadjuvant chemotherapy** |  |
| Yes | 12 (57) |
| No | 9 (43) |
| **Bladder-targeted therapy** |  |
| Radical cystectomy | 9 (43) |
| Radical cystectomy, aborted | 2 (10) |
| Radical cystectomy, declined | 1 (5) |
| Definitive radiotherapy | 2 (10) |
| None | 7 (33) |
| **Pathology** |  |
| pT0 | 0 (0) |
| pTis | 1 (5) |
| pT3 | 5 (24) |
| pT4 | 2 (10) |
| pN+ | 2 (10) |
| **Checkpoint inhibitor** |  |
| First line | 8 (38) |
| Second line | 12 (57) |
| Third line | 0 (0) |
| Fourth line | 1 (5) |
| **Site(s) of metastatic disease** |  |
| Retroperitoneal lymph node | 8 (38) |
| Peritoneal | 7 (33) |
| Pelvic (lymph node and soft tissue) | 4 (19) |
| Non-RPLN nodes | 3 (14) |
| Other soft tissue | 3 (14) |
| Osseous | 2 (10) |
| Lung | 1 (5) |
|  |  |
|  |  |
| PUC, plasmacytoid urothelial carcinoma; ICI, immune checkpoint inhibitor; RPLN, retroperitoneal lymph nodes | |
