# Supplementary material for: Natural history, response to systemic therapy, and genomic landscape of plasmacytoid urothelial carcinoma
Source: Br J Cancer. 2021 Jan 21;124(7):1214–21. doi: 10.1038/s41416-020-01244-2 (PMC8007750; doi:10.1038/s41416-020-01244-2)

## Slide 1
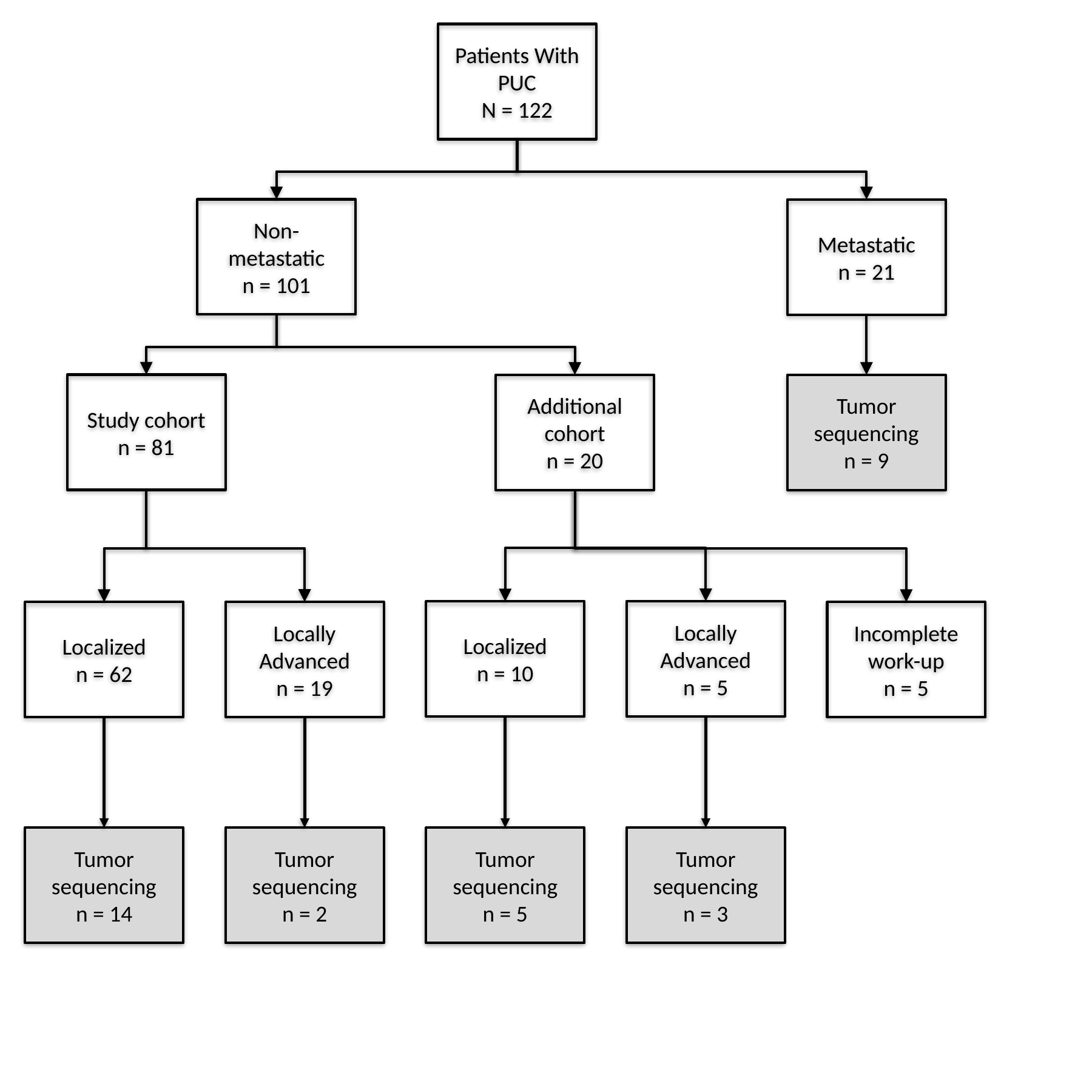

Patients With PUC
N = 122
Non-metastatic
n = 101
Metastatic
n = 21
Study cohort
n = 81
Additional cohort
n = 20
Tumor sequencing
n = 9
Locally Advanced
n = 5
Localized
n = 10
Locally Advanced
n = 19
Localized
n = 62
Incomplete work-up
n = 5
Tumor sequencing
n = 5
Tumor sequencing
n = 14
Tumor sequencing
n = 2
Tumor sequencing
n = 3

## Slide 2
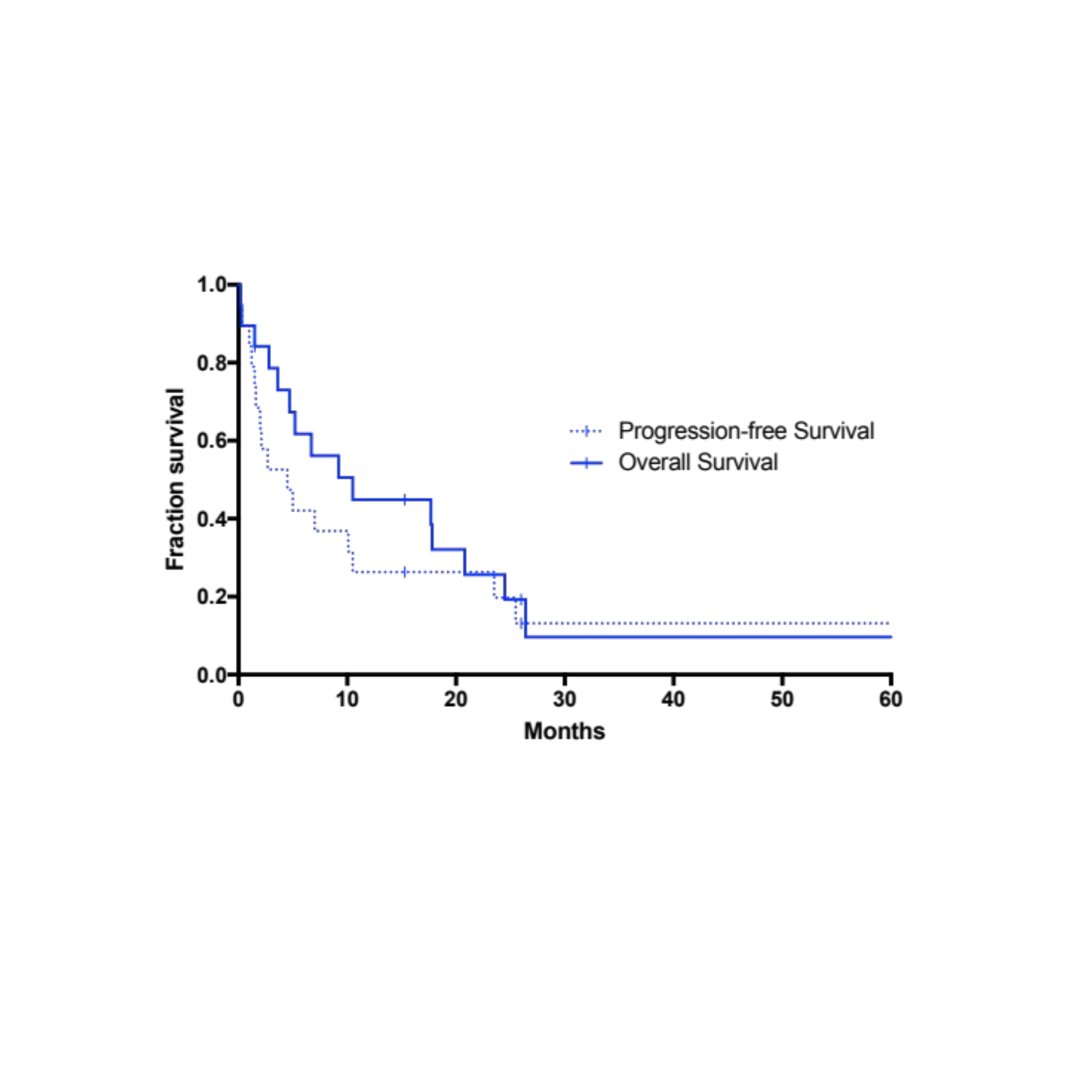

Supplement: Supplementary file 2 — Supplemental Figures 1 and 2 [file 41416_2020_1244_MOESM2_ESM.pptx]
